# *Echinometra lucunter* molecules reduce Aβ42-induced neurotoxicity in SH-SY5Y neuron-like cells: effects on disaggregation and oxidative stress

**DOI:** 10.1590/1678-9199-JVATITD-2023-0031

**Published:** 2023-12-01

**Authors:** Amanda Gomes da Silva, Mariana da Mata Alves, Admilson Aparecido da Cunha, Giovanna Arruda Caires, Irina Kerkis, Hugo Vigerelli, Juliana Mozer Sciani

**Affiliations:** 1 Integrated Pharmacology and Gastroenterology Unit (UNIFAG), Bragança Paulista, SP, Brazil.; 2 Laboratory of Genetics, Butantan Institute, São Paulo, SP, Brazil.; 3 Laboratory of Biochemistry and Biophysics, Butantan Institute, São Paulo, SP, Brazil.; 4 Center of Excellence in New Target Discovery, Butantan Institute, São Paulo, SP, Brazil.; 5 Laboratory of Natural Products, Postgraduate Program in Health Sciences, São Francisco University, Bragança Paulista, SP, Brazil.

**Keywords:** sea urchin, amyloid peptide 42, Alzheimer’s disease, protein clearance, oxidative stress

## Abstract

**Background:**

*Echinometra lucunter* is a sea urchin commonly found on America’s rocky shores. Its coelomic fluid contains molecules used for defense and biological processes, which may have therapeutic potential for the treatment of amyloid-based neurodegenerative diseases, such as Alzheimer's, that currently have few drug options available.

**Methods:**

In this study, we incubated *E. lucunter* coelomic fluid (ELCF) and fractions obtained by solid phase extraction in SH-SY5Y neuron-like cells to evaluate their effect on cell viability caused by the oligomerized amyloid peptide 42 (Aβ42o). Moreover, the Aβ42o was quantified after the incubation with ELCF fractions in the presence or not of cells, to evaluate if samples could cause amyloid peptide disaggregation. Antioxidant activity was determined in ELCF fractions, and cells were evaluated to check the oxidative stress after incubation with samples. The most relevant fraction was analyzed by mass spectrometry for identification of molecules.

**Results:**

ELCF and certain fractions could prevent and treat the reduction of cell viability caused by Aβ42o in SH-SY5Y neuron-like cells. We found that one fraction (El50) reduced the oligomerized Aβ42 and the oxidative stress caused by the amyloid peptide through its antioxidant molecules, which in turn reduced cell death. Mass spectrometry analysis revealed that El50 comprises small molecules containing flavonoid antioxidants, such as phenylpyridazine and dihydroquercetin, and two peptides.

**Conclusion:**

Our results suggest that sea urchin molecules may interact with Aβ42o and oxidative stress, preventing or treating neurotoxicity, which may be useful in treating dementia.

## Background

Animal venoms are rich sources of bioactive compounds, and many of them have neuroactive molecules, which can be used as pharmacological tools for the treatment of several disorders, including Alzheimer’s Disease, Parkinson’s Disease, Huntington’s Disease, amyotrophic lateral sclerosis, among others [1].

Alzheimer’s disease is the main type of dementia, characterized by extracellular plaques of amyloid peptides (Aβ), generated after precursor amyloid protein (APP) processing by beta and gamma-secretases, besides the formation of intraneuronal neurofibrillary tangles. Both features cause neurotoxicity and neurological damage in the affected area, which spreads through the brain, over time. Moreover, Aβ activates microglia to release pro-inflammatory mediators and reactive oxygen species [2]. Aβ has been detected in other neurological diseases, such as Parkinson’s [3], vascular dementia [4], Lewy body dementia [5], amyotrophic lateral sclerosis [6], and Down syndrome [7]. 

Molecules that reduce neurotoxicity by impairing amyloid plate formation or inducing degradation of amyloid peptides are being searched from animal venoms. Octovespin is one example. It is a peptide from *Polybia occidentalis* wasp venom, that was able to reduce the aggregation of Aβ in both *in vitro* and *in vivo* models [8]. Exenatide, developed for type 2 diabetes treatment, is now being evaluated for cell death induced by 6-hydroxydopamine (6-OHDA), Aβ, and oxidative stress agents [9]. Peptides from the sea anemone *Heteractis crispa* increased N2A viability, after exposure to 6-OHDA [10], and neuroprotection was observed by peptides from *Palythoa caribaeorum* in zebrafishes [11].

*Echinometra lucunter* is an abundant sea urchin living in America's intertidal rocky shore, which secretes several molecules for chemical defense and homeostasis [12, 13]. Our group has been studying secretion from *E. lucunter* spines and coelomic fluid and has described several biomolecules - small molecules, peptides, and proteins, important for the animal’s homeostasis. A cathepsin B/X is related to spine regeneration, a peptide from coelomic fluid participates in the innate immune system and a small molecule has pro-inflammatory activity, helpful against predators [14-16]. Thus, this animal can be used as a source of molecules therapeutically relevant.

In this study, we show molecules from *E. lucunter* sea urchin coelomic fluid (ELCF) that reduced the neurotoxicity caused by Aβ42 in SH-SY5Y neuron-like, in both preventive and treatment approaches.

## Methods

### Sample

*Echinometra lucunter* sea urchin was collected in São Sebastião SP, Brazil (23°49′53″S; 45°31′18″W), under license number 13852-1 from the Brazilian Environmental Agency (IBAMA), without distinction of sex, age or size. The coelomic fluid was extracted by puncturing the peristomial membrane and added to acetic acid 0.05%, in a proportion 40:1 v:v. The fluid was kept in an ice bath until further processing. The coelomic fluid (ELCF) was then centrifuged at 1248 × g for 5 min, at 4°C and the supernatant was processed by solid phase extraction (SPE) using C18 cartridges (Strata®, 55 µm, 70 Å, 5 g/20 mL, Phenomenex Inc., Torrance, CA, USA). The elution was performed with increased concentration of acetonitrile (0, 25, 50, 75, and 100%), in a solution containing 0.1% trifluoroacetic acid (TFA), generating fractions named El 0, 25, 50, 75, and 100, correspondent to the percent of acetonitrile used in the elution of compounds.

Aβ42 was purchased from FastBio (Ribeirão Preto, Brazil), manufactured by Biomatik (Canada), with 95.85% purity determined by HPLC and analyzed by mass spectrometry (the certificate of analysis is in additional file 1). The peptide was diluted in dimethyl sulfoxide (DMSO) to get a 1 mM solution, and then diluted in phosphate-saline buffer (PBS) 50 mM pH 7.2 to 100 µM. This solution (named Aβ42o) was maintained at 4°C for 24 hours for oligomerization. Before and after the dilution in PBS buffer, thioflavin-T was added and fluorescence value was obtained to confirm the oligomerization. This data is available in the additional file 2.

### Cell viability

SH-SY5Y cells (ECACC, Sigma Aldrich, St. Louis, MO, USA) were cultured in Dulbecco's Modified Eagle Medium: Nutrient Mixture F-12 (DMEM/F-12) (1:1) (Gibco Life Technologies, Grand Island, NY, USA) supplemented with 10% heat-inactivated fetal bovine serum (FBS) and 100 U/mL of penicillin/streptomycin (Gibco Life Technologies, Grand Island, NY, USA) in a humidified atmosphere of 5% CO_2_ at 37°C. In the 17^th^ passage, after adhesion, cells (1 × 10^4^ cells/well, 96 wells) were differentiated by adding 10 μM all-trans retinoic acid (Sigma Aldrich, Saint Louis, MO) in a media containing 1% FBS. This cell media was replaced every 2 days, until the 8^th^ day, when neuron-like cells were used in the experiments. After differentiation, ELCF was tested in a range of 0.1 to 100 µg/mL to test its potential to cause alteration in cell viability, as well as SPE fractions, tested in a concentration of 100 µg/mL. 

We used two approaches to verify if ELCF and its fractions interfere with the differentiated SH-SY5Y viability. (1) prevention: incubation of ELCF (10 µg/mL) or fractions for 1 or 6 h and then addition of Aβ42o (5 µM) for 48 hours; (2) treatment: incubation of Aβ42o (5 µM) for 48 hours and then addition of ELCF (10 µg/mL) or fractions for 3 or 24 hours. The treatments were performed in triplicate, with two independent experiments.

After treatment, the cell viability was determined by 3-(4,5-dimethylthiazol-2-yl)-2,5-diphenyltetrazolium bromide (MTT) assay, where the medium was removed, and the reagent was incubated for 3 hours in a concentration of 0.5 mg/mL. The blue formazan product was dissolved in DMSO, and the absorbance was measured at 540 nm. The results were plotted in a graph of % viable cells ± SEM, being the negative control (the same volume of sample, but PBS) the 100%, in a triplicate experiment, with two independent experiments.

For the treatment approach, a trypan blue exclusion test of cell viability was applied. Differentiated SH-SY5Y cells (1 x 10^4^ cells/well - 96 well plate) were treated with Aβ42o (5 µM) for 48 hours and then ELCF (10 µg/mL) for 24 h (control group received the same volume of PBS). After this period, the cell media was discarded, and cells were washed with PBS. Trypsin was added to remove cells from the plate, and the media was removed by centrifugation (1500 x g, 5 min). The trypan blue solution was added to the cells, which were placed in a Neubauer chamber to be counted in light microscopy. Viable cells (%) were determined as the total number of viable cells per mL divided by the total number of cells per mL, multiplied by 100.

### Identification and quantification of Aβ42 

The cell culture media was collected, and 60% acetonitrile in ultrapure water was added. The solution was centrifuged at 10000 rpm at 4 °C for 10 min. The supernatant was inserted into a C18 column (Titan, 80Å, 5 x 2.1 mm, 1.9 µm, Supelco) coupled to the mass spectrometry (QToF Xevo GS-XS, Waters Co., USA). Chromatography was performed by elution with acetonitrile containing 0.1% formic acid, in 3 steps: 0% B, 60% B, and 100% B, in a constant flow of 0.2 mL/min. The ions corresponding to Aβ42 (m/z 1129.0 and m/z 1505, to 4 and 3 charges respectively) as well as the fragments, generated after argon collision, were monitored after positive ionization, in a range of 300 to 1800 m/z and FWHM 40000 resolution at 500 m/z. For the MS/MS analysis. The instrument control and data acquisition were conducted by MassLinx 4.2.

Alternatively, 2 µL cell culture media collected from control (cells treated with PBS), cells treated with oligomeric Aβ42 and cells with Aβ42o+samples were added to 196 µL phosphate-saline (PBS) buffer 50 mM pH 7.2 and 2 µL thioflavin-T (ThT) 1 mM, in a 96-well plate. The fluorescence was read in λex = 365/λem = 405 nm and the results are shown as arbitrary units of fluorescence, as mean ± SEM of a triplicate, after being subtracted from a blank (ThT + PBS, at the same volume and concentration).

Thioflavin-T was also used to measure the oligomerization of Aβ42 in direct contact with ELCF and its fractions. Samples (10 µg/mL) were incubated with 50 mM pH 7.2 PBS buffer and 100 µM Aβ42o. ThT (1 mM) was added and after 5 minutes, the fluorescence was read in λex = 365/λem = 405 nm. Experiments were performed in triplicate and calculated as average ± SEM, being samples compared to a negative control (without Aβ42) and a positive control (Aβ42 without sample).

### Antioxidant activity

The antioxidant activity was assessed by the 2,2-Diphenyl-1-picrylhydrazyl (DPPH) and hydrogen peroxide (H_2_O_2_) methods, both conducted on a 96-well plate. 

For DPPH, ELCF and fractions (10 μg/mL) were diluted in 180 μL methanol and incubated with 0.1 mM DPPH reagent, diluted in methanol, in the dark. After 5 minutes at room temperature, the mixture had its absorbance read by a spectrophotometer at λ = 515 nm. 

For the hydrogen peroxide assay, samples were added to 0.1 M pH 7.0 phosphate buffer, containing 89 mM NaCl and hydrogen peroxide 0.2 mM. The mixture was incubated for 10 minutes at 37°C, and after that, 0.5 mL of HRPO (0.05 mg) and phenol (0.1 mg), diluted in 0.1 M pH 7.0 phosphate buffer, were added for 15 minutes at room temperature. Then, 1.3 M NaOH was added for 10 minutes, and the absorbance was read at λ = 610 nm.

As a positive control, ascorbic acid (1 mM) was used instead of samples for both assays. The % of oxidant activity was calculated by: [(Ac - As)/ Ac)] × 100, where Ac = absorbance of the control and As = absorbance of the test sample, being the control the reagent, without sample. The tests were performed in triplicate and calculated as average ± SEM. 

The antioxidant power of cells was measured using the Antioxidant Assay Kit (709001, Cayman Chemicals, MI, USA), following the manufacturer’s instructions. After being treated in the prevention or treatment approach, the cell culture media was collected and submitted to the test. The test was performed in triplicate and calculated as average ± SEM. 

### Compounds identification

The fraction El50 was analyzed by mass spectrometry for compound identification. The fraction was analyzed using reverse-phase ultraperformance liquid chromatography with a C18 column (1.7 µm, 100 Å, 2.0 mm × 50 mm). The elution was done using a binary gradient of 0-100% B over 40 min, being A = formic acid (FA)/H_2_O (1:1000) and B = FA/acetonitrile/H_2_O (1:900:100), at a constant flow rate of 0.2 mL/min. Mass spectrometry (Q-ToF Xevo GS, Waters Co.) was used for automatic monitoring of the column eluates in a positive ionization mode, with MS/MS analysis performed using argon collision energy. The scan was monitored in a range of 100 to 1800 m/z for MS and 50 to 1500 m/z for MS/MS, and FWHM 40000 resolution at 500 m/z. 

Equipment control and data acquisition were conducted using the MassLynx 4.2. Raw files were converted to mzML using MSConvert (ProteoWizard 3.0) and processed by the GNPS-MassIVE public data repository for untargeted MS^2^ data using compound identification and molecular networking with MS^2^ and spectral similarity (http://gnps.ucsd.edu). Data was set as 0.5 Da of precursor ion mass tolerance, 0.2 Da of fragment ion mass tolerance, 6 min matched peaks, and 0.7 score threshold. It was used all public spectra at GNPS, curated by the natural product scientific community [17].

### Statistical analysis

Data are presented as mean (SEM). Statistical analysis was performed using one-way ANOVA, with Tukey’s posttest, by comparing all groups, considering p value 0.05.

## Results

### Cell viability

**Figure 1.**
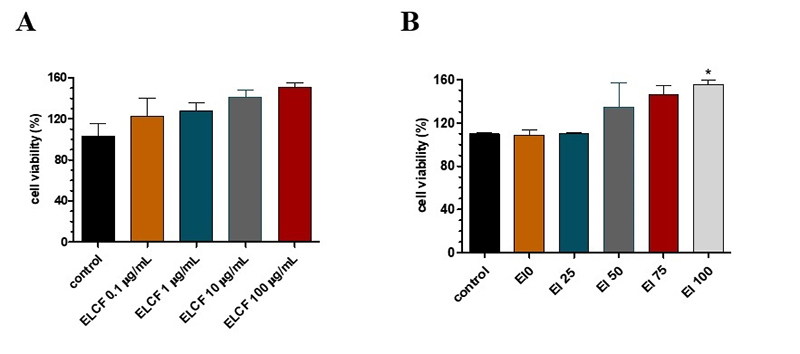
Cell viability of SH-SY5Y neuron-like after *Echinometra lucunter* coelomic fluid (ELCF) and its fractions. (**A**) Cell viability of different concentrations of coelomic fluid; (**B**) cell viability of *E. lucunter* coelomic fluid SPE fractions (100 µg/mL); Control = cells treated with PBS. Data are presented as mean (SEM). *indicates p < 0.05 of samples compared to control, by one-way ANOVA test, followed by Tukey’s test.

As shown in Figure 1A, ELCF did not cause any effect on the cell viability of neuron-like cells even at high concentrations. Similarly, the *E. lucunter* coelomic fluid fractions, which were generated after solid-phase extraction (Figure 1B), did not show any effect on cell viability. In fact, El 100 even increased cell viability.

**Figure 2.**
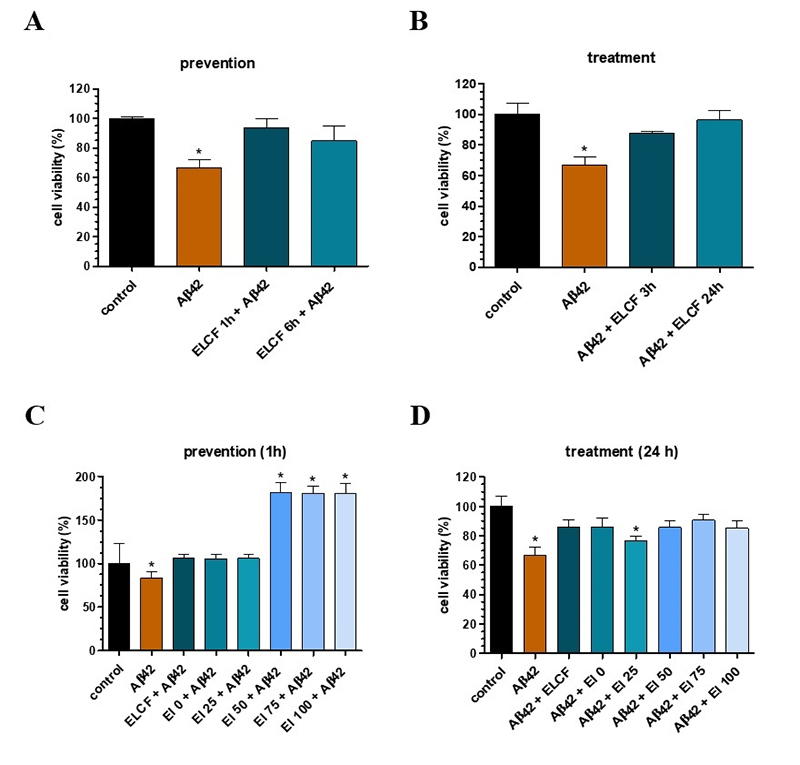
Cell viability of SH-SY5Y neuron-like after Aβ42o and *Echinometra lucunter* coelomic fluid (ELCF) and its fractions. (**A)** Prevention evaluation (addition of sample for 1 or 6h before the incubation of Aβ42o); (**B**) treatment evaluation (sample addition after the incubation of Aβ42o for 3 or 24h); (**C**) prevention evaluation of ELCF fractions; (**D)** treatment evaluation with ELCF fractions. Control = cells treated with PBS. Data are presented as mean (SEM). *indicates p < 0.05 of samples compared to control, by one-way ANOVA test, followed by Tukey’s test.

Afterward, ELCF was incubated in neuron-like cells to verify its ability to prevent or treat the reduction of cell viability, caused by the oligomerized amyloid peptide Aβ42. Aβ42o induced toxicity in neuron-like 48 h after incubation (Figure 2A, B, C, and D). ELCF, when was added for both 1 h and 6 h before the oAβ42, was able to prevent neurotoxicity (Figure 2>A). In a treatment effect, the addition of ELCF for 3 or 24h after Aβ42o incubation could reverse the toxic effect caused by the peptide (Figure 2B). 

Besides MTT, cell viability was quantified after trypan blue staining (additional file 3). The cell death caused by Aβ42o was confirmed (66% viable cells), as well as the effect of ELCF in reverting cell death (79% viable cells, statistically the same as the control group, 84% viable cells). The additional file 3 also shows a representative photo of the three conditions (control, Aβ42o, and Aβ42o followed by the treatment with ELCF), where is possible to see that Aβ42o caused a reduction in the number of cells, besides alteration in their morphology (cells not adhered, others adhered but without dendrites and with small cell body and nucleus with black spots). On the other hand, the treatment with ELCF reverted this altered morphology, and although the number of cells is smaller than the control group, the aspect is different from the Aβ42o group.

When we analyzed ELCF fractions, we could see interesting results in both prevention and treatment effects. All fractions prevented the reduction of cell viability caused by Aβ42o, and El50, 75, and 100 significantly increased this viability (Figure 2C). Regarding treatment, only fraction El25 could not reverse the toxic effects caused by Aβ42o, while other fractions reproduced the observed effects of the raw sample (Figure 2D).

### Aβ42 identification and quantification

Mass spectrometry analysis was used to verify the presence of monomeric amyloid peptide in the cell culture media, after the incubation of samples in both prevention and treatment approaches. The ion corresponding to Aβ42 (1129.0 or m/z 1505) was absent in cells without any treatment (Table 1, control) and present in cells in which the peptide was added (Table 1, Aβ42). When ELCF and El 0 or El 25 fractions were incubated in the prevention approach, the ions were not identified in the media (Table 1, prevention), while they were observed with El 50, 75, and 100. When ‘treatment samples’ were analyzed, the ion was identified in all samples, indicating that the monomeric amyloid peptide was in the cell media. Mass spectra are shown in additional file 4.

**Table 1.** Presence (+) or absence (-) of Aβ42 ion (m/z 1129.0 or m/z 1505) in the culture media of SH-SY5Y neuron-like treated with the amyloid peptide*, E. lucunter* coelomic fluid and its SPE fractions before (1 h = prevention) or after (24 h = treatment).

	Prevention	Treatment
Control	-	-
Aβ42	+	+
ELCF	-	+
El 0	-	+
El 25	-	+
El 50	+	+
El 75	+	+
El 100	+	+

**Figure 3.**
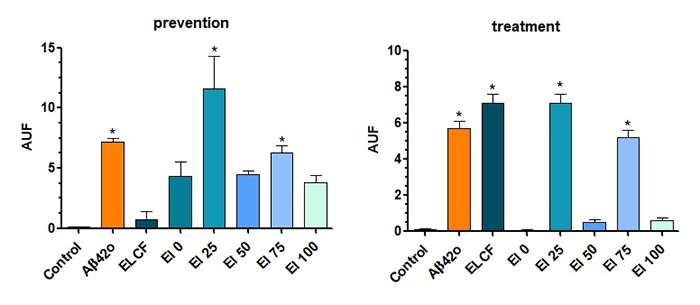
Percent of aggregated Aβ detected by fluorescence, in the presence of thioflavin-T, in SH-SY5Y neuron-like culture media treated with Aβ, *Echinometra lucunter* coelomic fluid (ELCF) and its fractions, obtained by solid-phase extraction. Prevention = samples incubated 1 h before Aβ42o; treatment = samples incubated 24h and then Aβ42o. Data are presented as mean (SEM). *indicates p < 0.05 of samples compared to control, by one-way ANOVA test, followed by Tukey’s test.

Fluorescence emission analysis of the cell culture media was performed after incubation with thioflavin-T to detect Aβ42 in its oligomeric form. We observed that ELCF and fractions El 0, 50, and 100 were effective in reducing Aβ42o levels in the prevention approach and the same fractions in the treatment approach (Figure 3).

**Figure 4.**
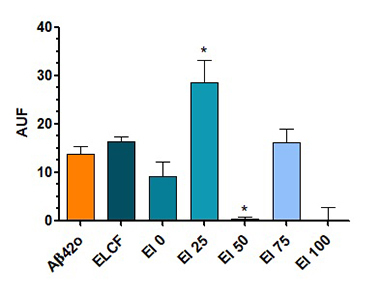
Percentage of aggregated Aβ detected by fluorescence, in the presence of thioflavin, in *Echinometra lucunter* coelomic fluid and its fractions added to Aβ42. Data are presented as mean (SEM). *indicates p < 0.05 of samples compared to control, by one-way ANOVA test, followed by Tukey’s test.

To verify if the reduction of Aβ42 oligomerization is caused by the direct action of ELCF and its fractions, we added the oligomerized amyloid peptide to each fraction and then incubated thioflavin-T. We could see that ELCF did not cause any effect, but when fractions were evaluated, El 50 and 100 reduced the oligomerization of amyloid peptide (Figure 4), in agreement with the previous results of Aβ42 oligomerization, observed in the cell media.

### Oxidative stress

**Figure 5.**
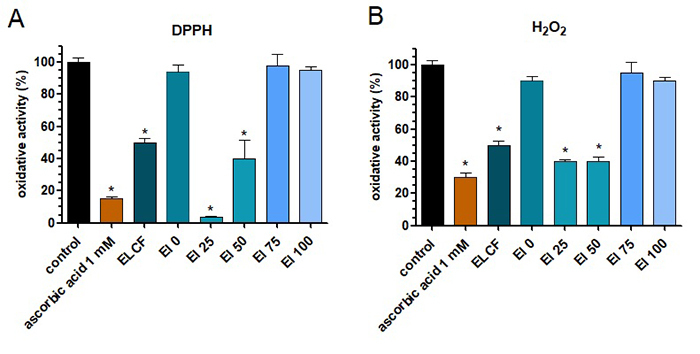
Oxidative activity of *Echinometra lucunter* coelomic fluid (ELCF) and its fractions. (**A**) DPPH assay; (**B**) peroxide assay. Control = reagent without samples; ascorbic acid is the positive control (antioxidant). Data are presented as mean (SEM) *indicates p < 0.05 of samples compared to control, by one-way ANOVA test, followed by Tukey’s test.

*E. lucunter* coelomic fluid and its fractions were tested to verify the presence of antioxidant molecules by DPPH and peroxide assays. It was verified that the coelomic fluid, El 25 and 50 were able to reduce the oxidant effect of the reagent, acting as an antioxidant, as well as ascorbic acid, used as a positive control (Figures 5A and B).

**Figure 6.**
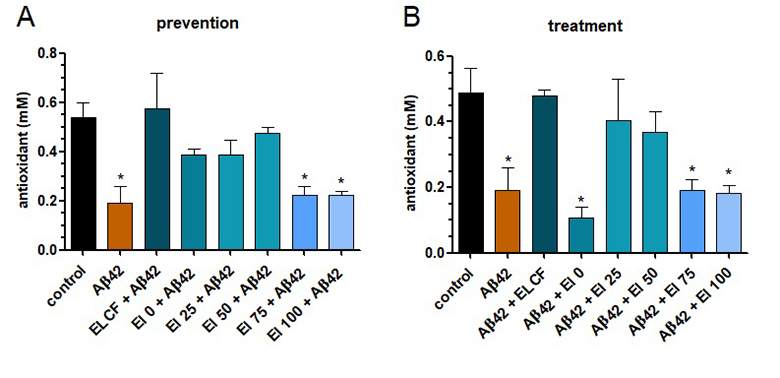
Antioxidant power of SH-SY5Y neuron-like media after treatment with Aβ42o or *Echinometra lucunter* coelomic fluid or its fractions, obtained by solid phase extraction. (**A**) Prevention approach = samples incubated 1 h before Aβ42o; (**B**) treatment approach = samples incubated 24h and then Aβ42o. Data are presented as mean (SEM). *indicates p < 0.05 of samples compared to control, by one-way ANOVA test, followed by Tukey’s test.

The cell culture media from SH-SY5Y neurons-like, previously treated with ELCF or its fractions, was collected for antioxidant capacity assay. It was observed that Aβ42o reduced the antioxidant power for both prevention and treatment approaches, compared to a control group, with neurons treated with PBS. On the other hand, *E. lucunter* coelomic fluid presented more antioxidants, in mM, than Aβ42, similarly to the control group. The fraction El 25 was able to reproduce these effects, while the others diminished antioxidant power, as shown in Figure 6A (prevention) and B (treatment).

### Compounds identification

Due to its interesting effect of preventing or reversing cell death caused by Aβ42o, through a mechanism of reducing oligomerization and controlling oxidative stress, fraction El 50 was analyzed by mass spectrometry to determine its composition. After comparing the mass spectra to the GNPS database, it was possible to identify six small molecules (Table 2), besides two peptides previously identified by our group, with sequences LLHA and AAPCPDVEVSEQF, corresponding to this fraction [12]. No proteins were detected in this sample (data not shown).

**Table 2.** Compound identified in GNPS database.

Compound name	Spectrum ID	Exact mass	Precursor m/z
Taxifolin (dihydroquercetin)	CCMSLIB00006410892	304.058	305.07
Benzalkonium chloride (C12)	CCMSLIB00000531495	304.29	304.29
C17-Sphinganine	CCMSLIB00009943587	287.50	288.276
DAPG	CCMSLIB00004679387	210.053	211.061
Minaprine	CCMSLIB00003137768	298.179	299.188
phenazine-1-carboxylic acid	CCMSLIB00000839196	224.059	225.066

## Discussion

The amyloid cascade hypothesis is the most acceptable to explain Alzheimer’s disease, in which APP protein, present in neurons, is cleaved by secretases, generating amyloid peptides [2]. The Aβ40 is the most abundant amyloid peptide (80 to 90%), but the Aβ42 is the most toxic, due to its hydrophobic nature, which allows plaque formation and deposit into the neurons [18]. That is the reason we chose to study Aβ42 instead of other amyloid peptides, and we could verify its neurotoxicity in our cell model, the SH-SY5Y cell line. This line has been widely used for neurodegenerative diseases after differentiation with agents, such as retinoic acid, that changes its morphology to be similar to primary neurons, with neuritic process, electrical excitability, and synaptophysin-positive synapses, characteristics of cholinergic and dopaminergic neurons [19]. 

Using this model, we demonstrated that *E. lucunter* coelomic fluid prevents an impaired reduction of cell viability induced by oligomeric amyloid peptide 42. Some fractions could reproduce such effects - fractions El 50, 75, and 100. Cell viability was estimated by MTT assay, which reflects the metabolic activity of the cells and therefore is an indirect measure of cell death. Although is a widely used method to estimate cell viability, we could confirm our first results using trypan blue staining. Moreover, the percent of viable cells observed here after Aβ42 incubation was compatible with the literature. 

At physiologically relevant concentrations, monomeric Aβ is not associated with cellular toxicity. However, soluble oligomers, which exhibit considerable heterogeneity in terms of size and structure, have been demonstrated to have significant neurotoxicity [20]. The oligomers induce synaptic dysfunction and neuron apoptosis, besides inducing oxidative stress and the release of inflammatory mediators, contributing to the amyloid-based neurodegenerative disease progression [21, 22]. 

Considering the description of animal venoms in reducing peptide amyloid and the presence of antioxidative molecules in sea urchins, we studied these two mechanisms, to understand how ELCF and fractions reduced the toxicity caused by Aβ42o: amyloid peptide removal from the cells in its monomeric or aggregated form, and the reduction of oxidative stress. For that, we opted to work with fractions, instead of the raw fluid, to reduce the number of molecules and get an assertive response.

Fractions El 25 and 75 were not able to reduce the oligomerized Aβ42 in both cellular mechanisms and by direct action on the peptide. Regarding fraction 25, only the oligomerized form of the peptide was found, which explains the lack of effect on either prevention or treatment approach on cell death.

On the other hand, fractions El 50 and 100 were effective in reducing oligomerized Aβ42 levels in cell media, in both prevention and treatment approaches, as evidenced by ThT quantification, a reagent that binds only to oligomeric amyloid peptides. While oligomeric peptides were still detected in the preventive approach, the results were more pronounced in the treatment with *E. lucunter* fractions 0, 50, and 75, suggesting that the molecules act on the oligomeric peptides after their formation without affecting the aggregation process.

Our findings confirmed that these three fractions were able to reduce the oligomers even in the absence of cells, indicating a direct action on the oligomer structure. Therefore, one mechanism of reduction of amyloid peptide toxicity by fractions El 50 and 100 is the disaggregation of oligomeric amyloid peptide, especially in a treatment approach, making it less toxic to neurons [23].

Using this same ThT assay, molecules isolated from venoms presented similar results. Camargo et al. [8] verified that a synthetic peptide optimized from wasp venom was able to prevent Aβ aggregation, and consequently reduced the toxicity caused by the amyloid peptide, confirmed by animal’s tests, in which memory impairment was reversed. Moreover, one study showed that polyphenols inhibited the oligomerization of amyloid peptides, as we showed here, with a mechanism of monomer stabilization or oligomer disaggregation [24]. Epigallocatechin gallate and myricetin are structures similar to the one found here (in El 50), with antiaggregating effects on Aβ peptide [25]. The venom from the scorpion *Buthus martensii Karsch*, containing peptides resistant to heat, increased neurogenesis and, in a *Caenorhabditis elegans* model that expresses Aβ1-42, reduced Aβ plaque deposition, in comparison to an untreated group [26]. 

Most of the evidence suggests that the N-terminal and β1 regions of Aβ are crucial in disrupting the aggregation process and controlling the toxicity of stabilized oligomers. Changes in the recognition of the monomer/membrane are associated with alterations in the accessibility of the hydrophobic β1-turn region and charged N-terminus, a probable site of inhibitory molecules, and this is one mechanism of disaggregation and reduction of toxicity by oligomers [27].

The exact mechanism of amyloid plaque removal or oligomerization reduction observed here needs to be further confirmed and studied, using other techniques than ThT, such as electronic microscopy or mass spectrometry focused on proteins [28, 29]. 

Another mechanism demonstrated here was the oxidative stress caused by Aβ42 and its reversion by *E. lucunter* samples. We observed that the *E. lucunter* coelomic fluid and fractions El 25 and 50 have intense antioxidant propriety, evaluated by two assays. When these samples were incubated in neuron-like cells, they could increase the antioxidant power in SH-SY5Y-treated cell culture media, in both prevention and treatment approaches, indicating reactive oxygen species (ROS) removal.

Antioxidants have already been described in sea urchins. The first report was from McClendon, in 1912, in which it was reported an antioxidant effect in the coelomic fluid from *Arbacia punctulate* [30]. A pigment from the *Anthocidaris crassispina* shell showed important antioxidant activity by the DPPH method [31]. Pigments derived from naphtoquinone, with high antioxidant activity, were isolated from *Echinometra mathaei*, a sea urchin species related to *Echinometra lucunter*, used in this work [32].

Echinochromes were described in sea urchins, being the most abundant Echinochrome A, clinically used in cardiology and ophthalmology, without causing adverse effects [33]. Several free radicals scavenging mechanisms have been related to this molecule [34] 

Sintsova et al. [10] showed similar results: peptides from *Heteractis crispa* sea anemone reduced reactive oxygen species (ROS) production in Neuro-2A cells, induced by 6‐OHDA, due to the ability of molecules to scavenge free radicals. 

Antioxidants are molecules that scavenge free radicals, able to restore mitochondrial damage. Mitochondrial abnormalities, such as the generation and release of ROS, cause lipid and protein peroxidation, and DNA damage. Oxidative stress and consequent neuron apoptosis have been observed in Alzheimer’s Disease, closely related to Aβ deposition [35, 36]. The release of inflammatory mediators caused by Aβ also induces the production of ROS. Thus, the control of oxidative stress would impair the toxicity caused by Aβ42, which we observed in this work.

Taxifolin, a molecule identified in El 50, belongs to the flavanonols class and possesses powerful antioxidant properties [37], which may be responsible for controlling the oxidative stress observed in this study. 

Other identified molecules could have important biological effects. Phenazine-1-carboxylic acid, an aromatic carboxylic acid, has antimicrobial and antifungal activity, which could be important for the maintenance of coelomic fluid in sea urchins and has already been found in jellyfish [38]. Minaprine is a phenylpyridazine, used for the treatment of depression due to its action as a reversible inhibitor of MAO-A, serotonin, and dopamine uptake inhibitor. Moreover, it has been found that minaprine weakly inhibits acetylcholinesterase, being considered for Parkinson’s Disease [39].

## Conclusion

We identified a fraction (El50) from *E. lucunter* coelomic fluid able to prevent and reverse the reduction of cell viability caused by oligomerized Aβ42 by its disaggregation, besides the reduction of oxidative stress. Further experiments are necessary to understand the exact mechanism of oligomer reduction, and pathways that would prevent cell death, but these two mechanisms are relevant for amyloid peptide-related diseases, such as Alzheimer’s, which have few options for treatment.

## Supplementary material

The following online material is available for this article:

Additional file 1. Certificate of analysis.

Additional file 2. Oligomerization of Aβ42 peptide after dilution to PBS buffer at 4°C for 24 hours, measured by arbitrary units of fluorescence (AUF) after staining with Thioflavin-T.

Additional file 3. Representative images of differentiated SH-SY5Y cell culture (A) without any treatment, (B) after incubation of 5 µM of Aβ42o and (C) after treatment with ELCF of cells exposed to 48h Aβ42o. The image suggests a reduction in the number of cells after treatment with both Aβ42o and ELCF, the number of cells was reduced compared to control. However, in B the morphology of cells is altered, in agreement with the MTT assay. In D, trypan blue staining of cells for counting and determination of viable cells.

Additional file 4. Mass spectra of culture media from SH-SY5Y cell culture. (A) Control, without treatment; (B) cells treated with 5 µM Aβ42o, where is possible to see ions 1129 and 1505 m/z, correspondent to the amyloid peptide; (C) cells treated with ELCF and then 5 µM Aβ42o (prevention approach), where no Aβ42o ions were detected; (D) cells treated with 5 µM Aβ42o and then ELCF (treatment approach), where is possible to see the ion 1129 m/z (arrow in the zoomed image).
