# Supplementary material for: Echinometra lucunter molecules reduce Aβ42-induced neurotoxicity in SH-SY5Y neuron-like cells: effects on disaggregation and oxidative stress
Source: J Venom Anim Toxins Incl Trop Dis. 2023 Dec 1;29:e20230031. doi: 10.1590/1678-9199-JVATITD-2023-0031 (PMC10694836; doi:10.1590/1678-9199-JVATITD-2023-0031)
Supplement: Additional file 1. [file 1678-9199-jvatitd-29-e20230031-s1.pdf]

J Venom Anim Toxins incl Trop Dis

https://doi.org/10.1590/1678-9199-JVATITD-2023-0031

Supplementary Material to "*Echinometra lucunter* molecules reduce Aβ42-induced neurotoxicity in SH-SY5Y neuron-like cells: effects on disaggregation and oxidative stress"

Additional file 1. Certificate of analysis.

BIOMATIK

Biomatik
Tel: (519) 489-7195, (800) 836-8089
Fax: (519) 231-0140, (877) 221-3515
Email: info@biomatik.com
http://www.biomatik.com

Certificate of Analysis

**Date:**	2021-07-21
**Order Number:**	#SP210838
**Product Type:**	Chemically synthesized peptide
**Catalog Number:**	180231
**Peptide Name:**	Ab 42
**Sequence (N to C):**	DAEFRHDSGYEVHHQKLVFFAEDVGSNKGAIIGLMVGGVVIA
**MW:**	4514.14
**Salt Form:**	Trifluoroacetate (TFA Salt)
**Quantity:**	5.0mg
**Suggested Solvent:**	1.0mg peptide soluble in 1.0ml DMSO
**Lot Number:**	P210714-SY180231
**Appearance:**	White to off-white lyophilized powder.
**Storage:**	Store lyophilized peptide at -20°C upon receipt. Reconstitute only the amount of peptide needed for immediate use.
**Limited Usage:**	For Research Use Only. Not for use in diagnostic procedures, or for administration to humans or animals.

**ASSAY**	**SPECIFICATION**	**ACTUAL**
**MW by MS**	4514.00	Conforms
**Purity by HPLC**	>95%	95.85%
**Peptide Content**	N/A	N/A
**TFA Content**	N/A	N/A
**Moisture Content**	N/A	N/A

Integrated Products & Services for Life Sciences

BIOMATIK

Biomatik
Tel: (519) 489-7195, (800) 836-8089
Fax: (519) 231-0140, (877) 221-3515
Email: info@biomatik.com
http://www.biomatik.com

MS REPORT

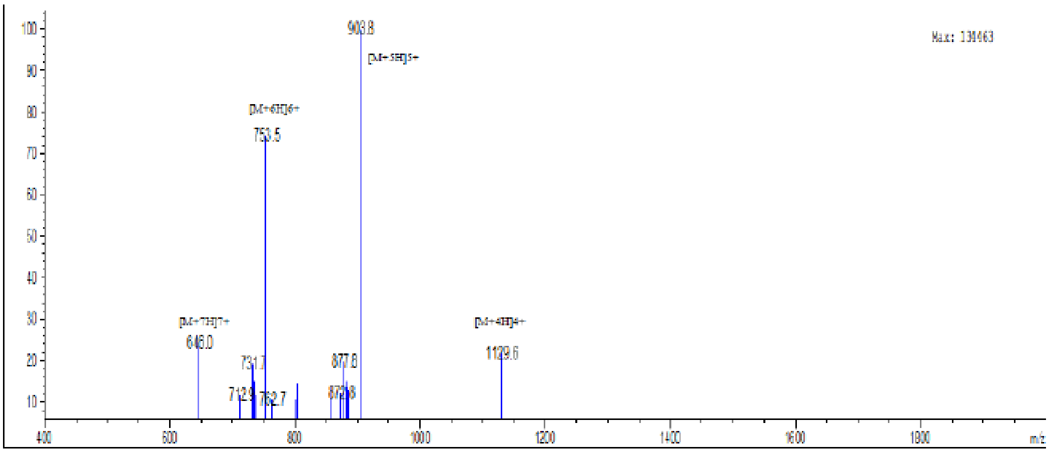

Sample Description		Instrument	Agilent-6125B		
Analyzed date:	2021-07-15	Probe:	ESI	Probe Bias:	+ 4.5kv
Analyst:	YU	Nebulizer Gas Flow:	1.5L/min	Detector:	1.5kv
Sample:	Ab42 DA-42	CDL:	-20.0v	T. Flow:	0.2ml/min
M.W.:	4514.14	CDL Temp.:	250 ℃	B. Conc.:	50%H2O/50%ACN
Lot. No.:	P210714-SY180231	Block Temp.:	200 ℃		

Biomatik
Tel: (519) 489-7195, (800) 836-8089
Fax: (519) 231-0140, (877) 221-3515
Email: info@biomatik.com
http://www.biomatik.com

HPLC REPORT

Structure : Ab42 DA-42
Number : 010250011
Lot No : P210714-SY180231
Column : 4.6×250mm,PLRP-S 100A
Solvent A : 0.1% trifluoroacetic in 100% acetonitrile
Solvent B : 0.1% trifluoroacetic in 100% water
Gradient :

	A	B
0.01min	10%	90%
25min	100%	0%
25.1min	100%	0%
30min	STOP	

Flow rate : 1.0 mL/min
Wavelength : 220nm
Volumn : 5ul

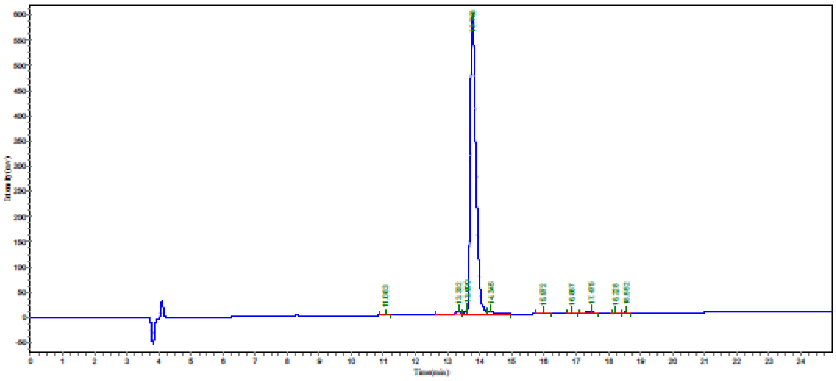

Peak No.	Ret Time	Height	Area	Conc..
1	11.063	1195.693	9867.498	0.1311
2	13.332	6196.430	81125.711	1.0778
3	13.600	9062.938	45557.531	0.6052
4	13.763	586355.000	7215245.500	95.8550
5	14.345	5972.477	90001.203	1.1957
6	15.972	1369.761	11004.504	0.1462
7	16.867	866.338	7008.294	0.0931
8	17.475	3994.174	48800.383	0.6483
9	18.228	1337.843	9053.433	0.1203
10	18.552	1335.607	9582.392	0.1273
Total				100.0000

Integrated Products & Services for Life Sciences

Additional file 2

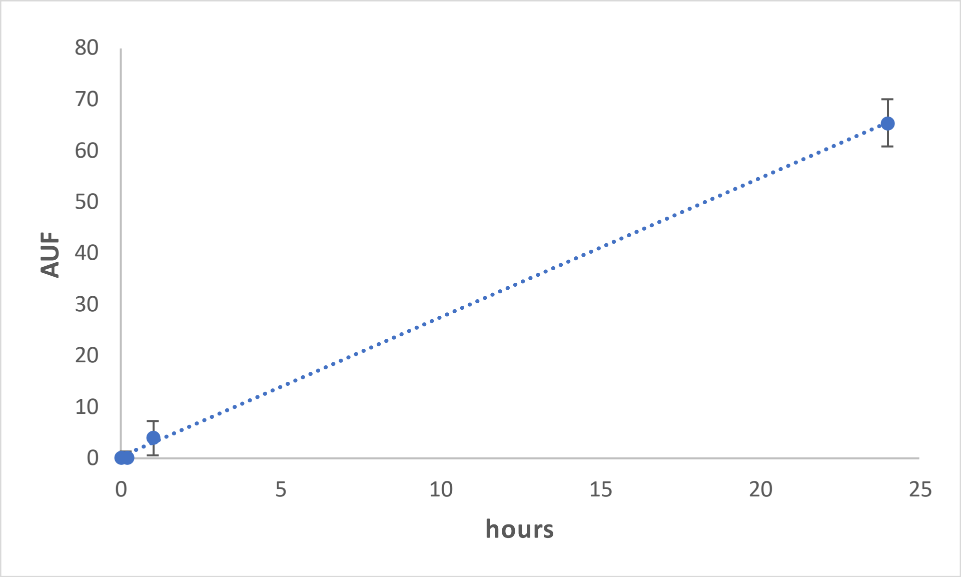

Oligomerization of Aβ42 peptide after dilution to PBS buffer at 4°C for 24 hours, measured by arbitrary units of fluorescence (AUF) after staining with Thioflavin-T.

Additional file 3

A B C

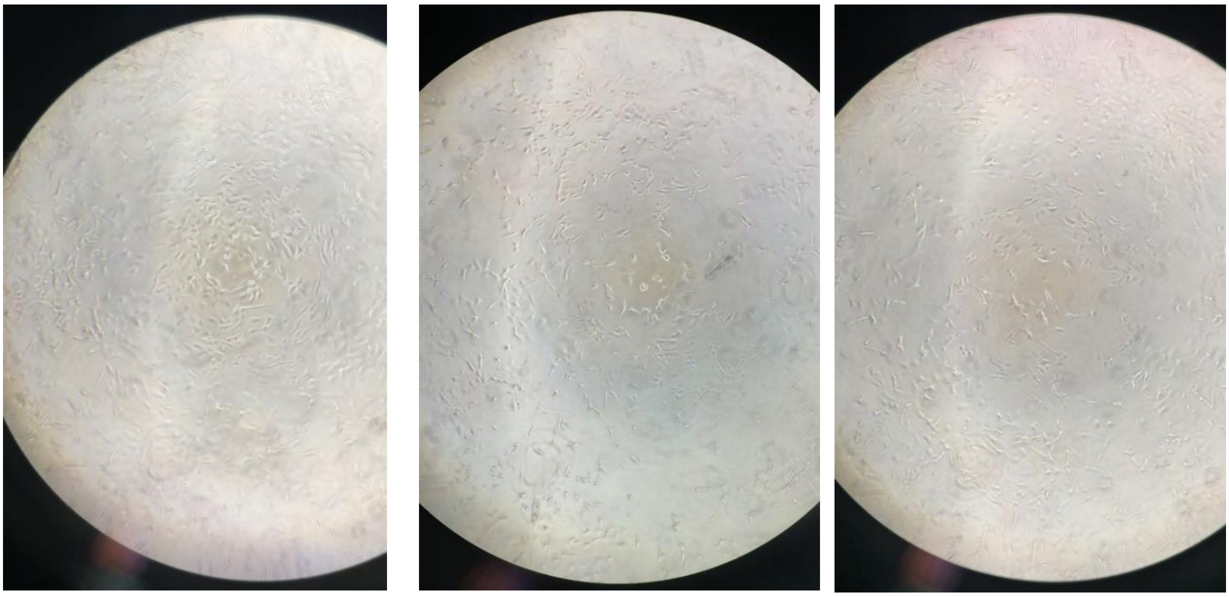

D

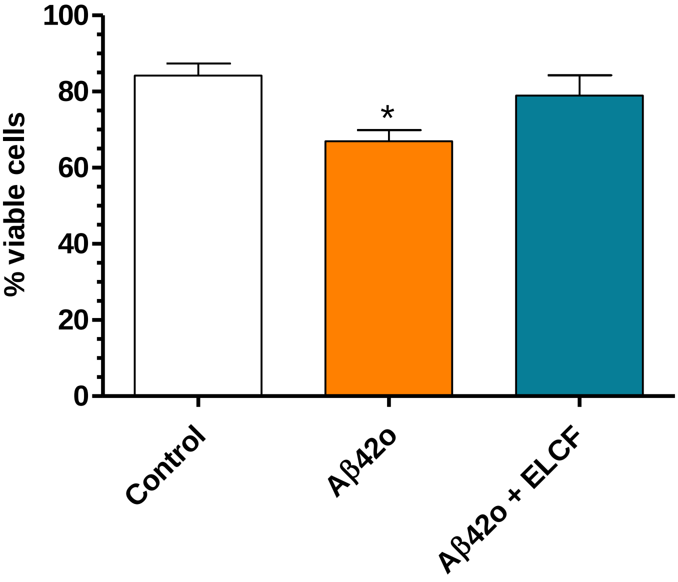

Representative images of differentiated SH-SY5H cell culture (A) without any treatment, (B) after incubation of 5 μM of Aβ42o and (C) after treatment with ELCF of cells exposed to 48h Aβ42o. The image shows that after treatment with both Aβ42o and ELCF, the number of cells was reduced compared to control. However, in B the morphology of cells is altered, in agreement with the MTT assay. In D, trypan blue staining of cells for counting and determination of viable cells.

Additional file 4

A

Control

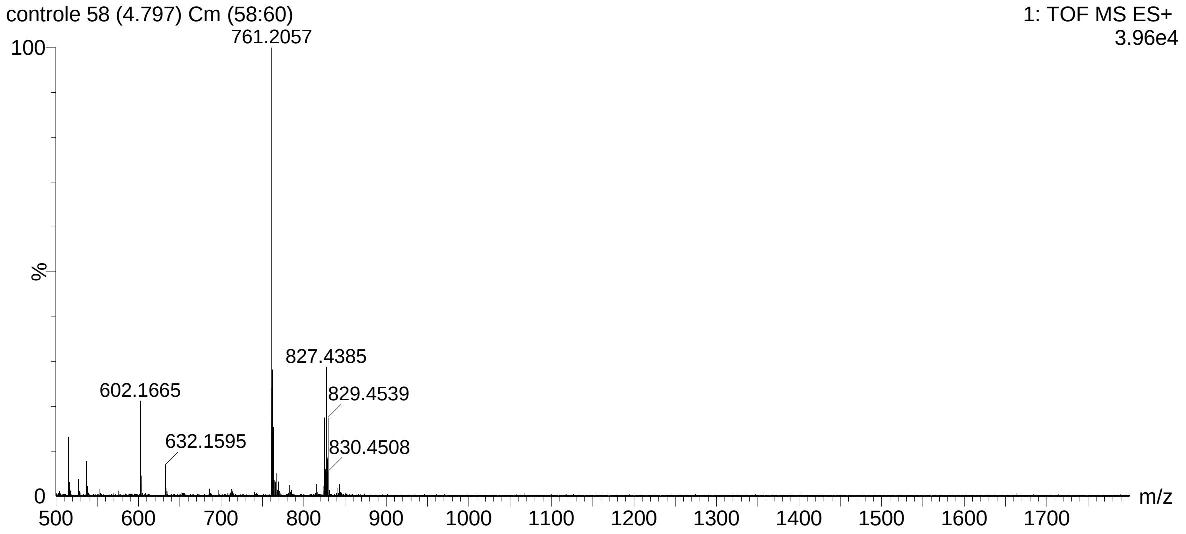

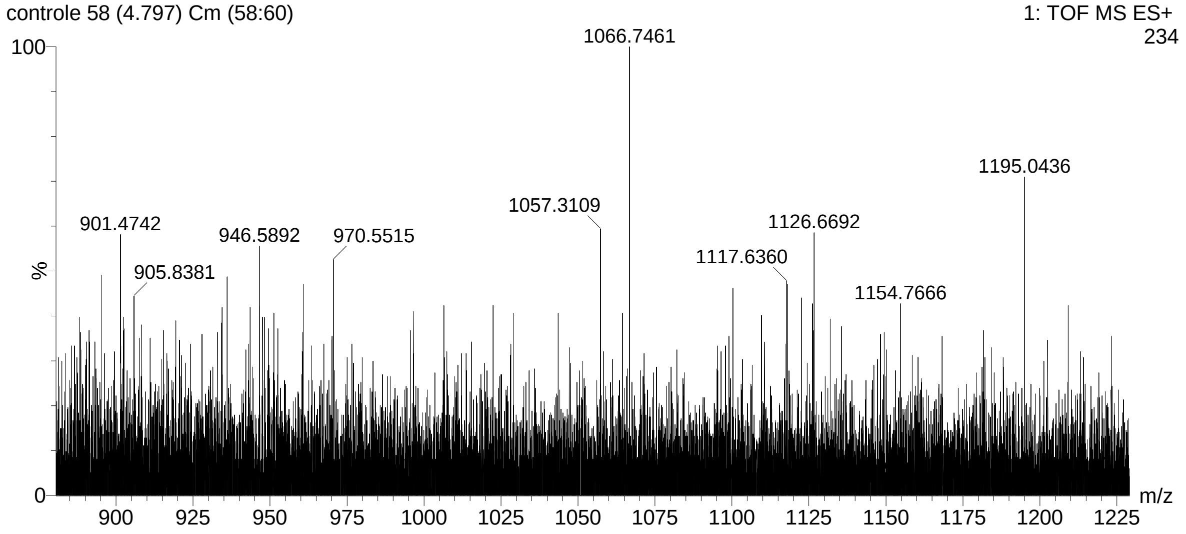

B

Aβ42

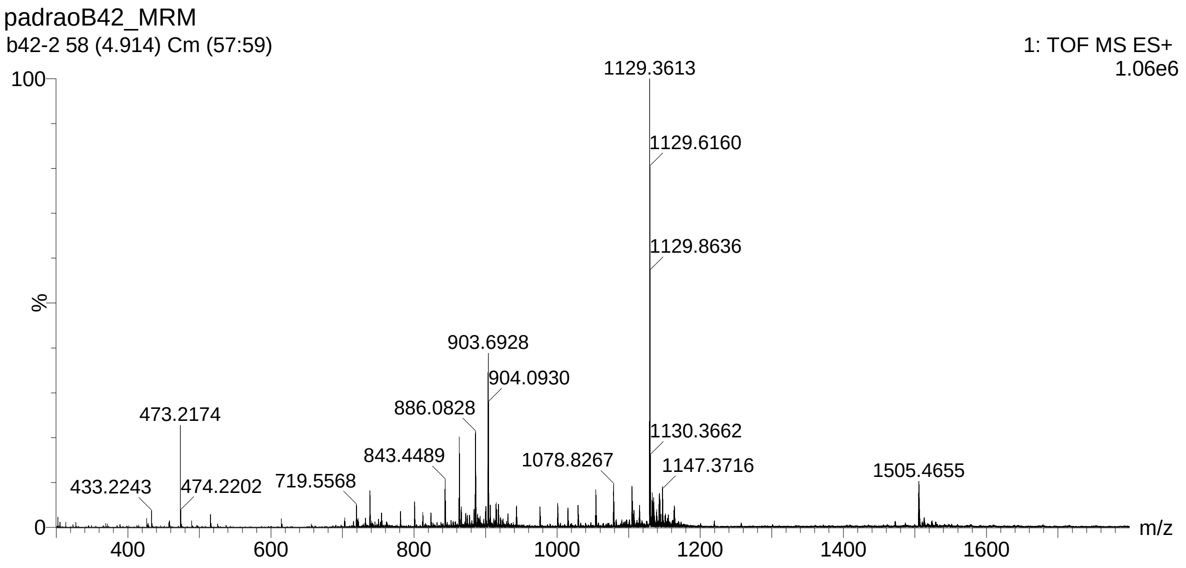

C

ELCF – prevention approach

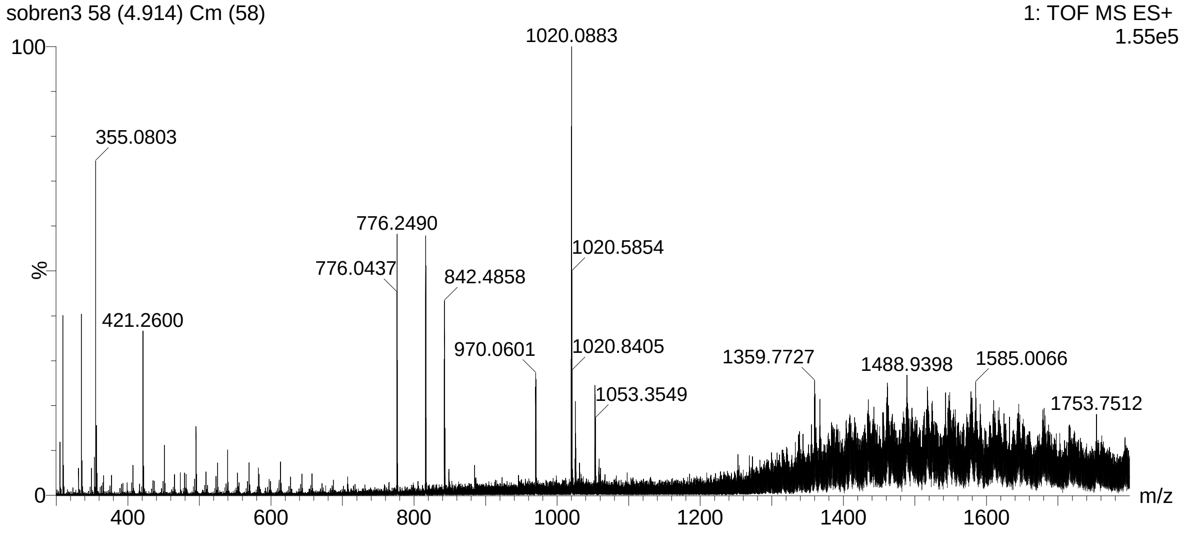

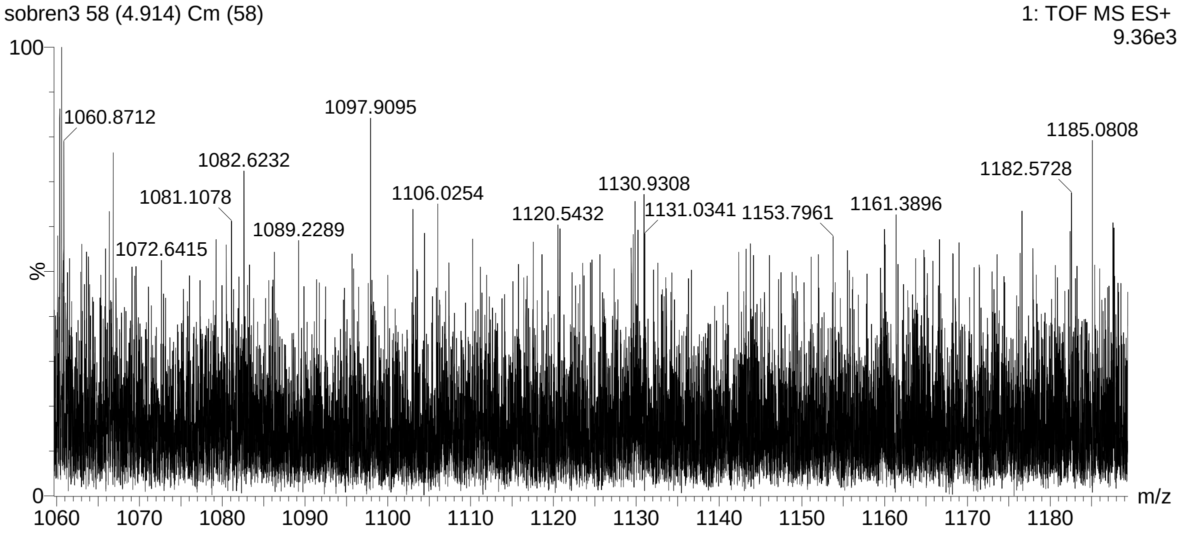

D

ELCF – treatment approach

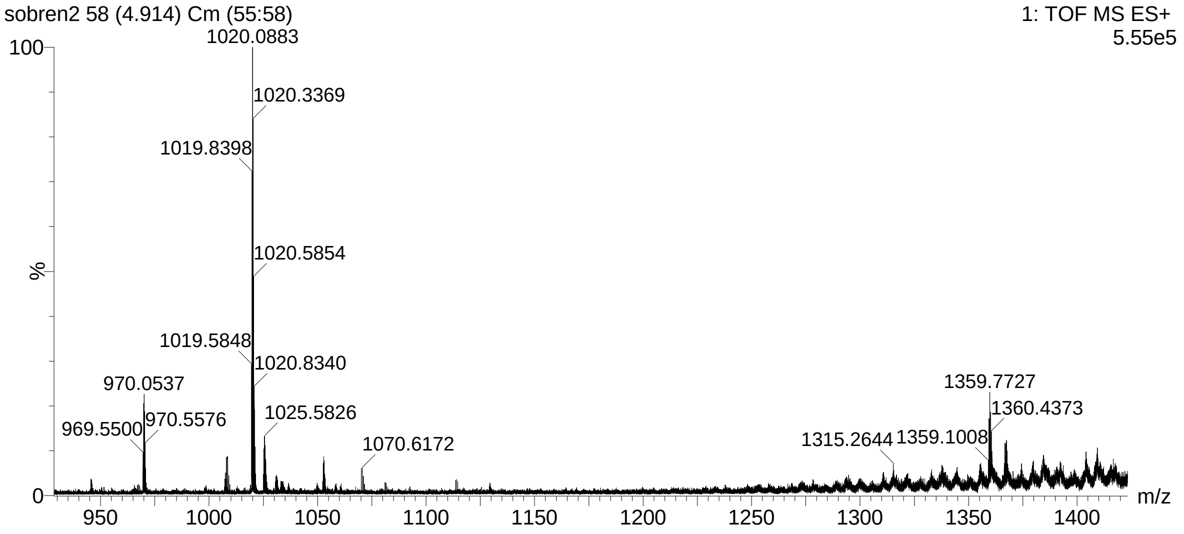

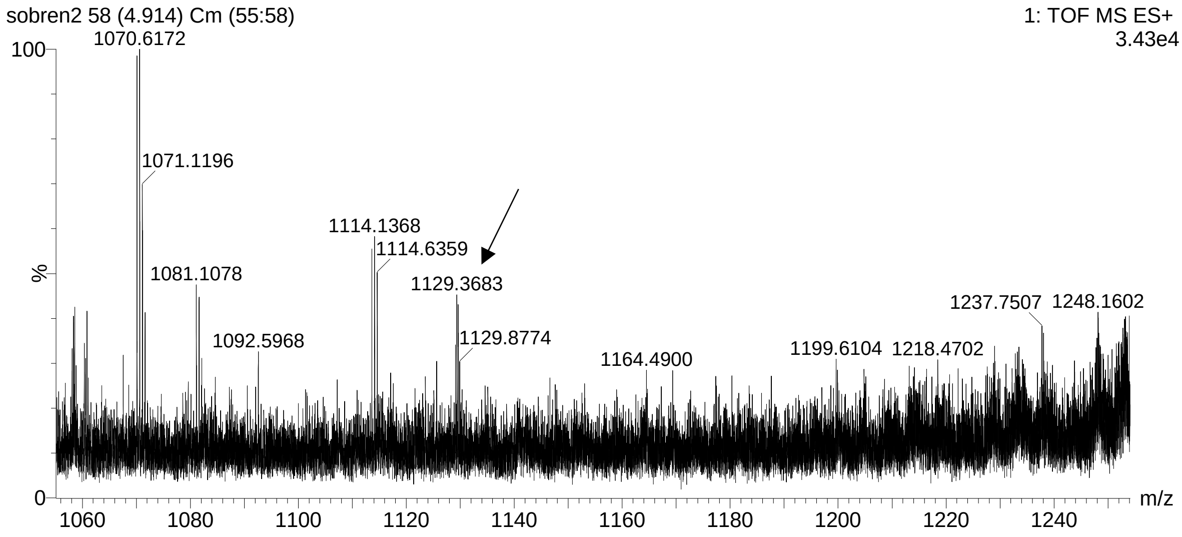

Mass spectra of culture media from SH-SY5Y cell culture. (A) Control, without treatment; (B) cells treated with 5 µM Aβ42o, where is possible to see ions 1129 and 1505 m/z, correspondent to the amyloid peptide; (C) cells treated with ELCF and then 5 µM Aβ42o (prevention approach), where no Aβ42o ions were detected; (D) cells treated with 5 µM Aβ42o and then ELCF (treatment approach), where is possible to see the ion 1129 m/z (arrow in the zoomed image).
