# Supplementary material for: Echinometra lucunter molecules reduce Aβ42-induced neurotoxicity in SH-SY5Y neuron-like cells: effects on disaggregation and oxidative stress
Source: J Venom Anim Toxins Incl Trop Dis. 2023 Dec 1;29:e20230031. doi: 10.1590/1678-9199-JVATITD-2023-0031 (PMC10694836; doi:10.1590/1678-9199-JVATITD-2023-0031)
Supplement: Additional file 2. [file 1678-9199-jvatitd-29-e20230031-s2.pdf]

J Venom Anim Toxins incl Trop Dis

https://doi.org/10.1590/1678-9199-JVATITD-2023-0031

Supplementary Material to "*Echinometra lucunter* molecules reduce Aβ42-induced neurotoxicity in SH-SY5Y neuron-like cells: effects on disaggregation and oxidative stress"

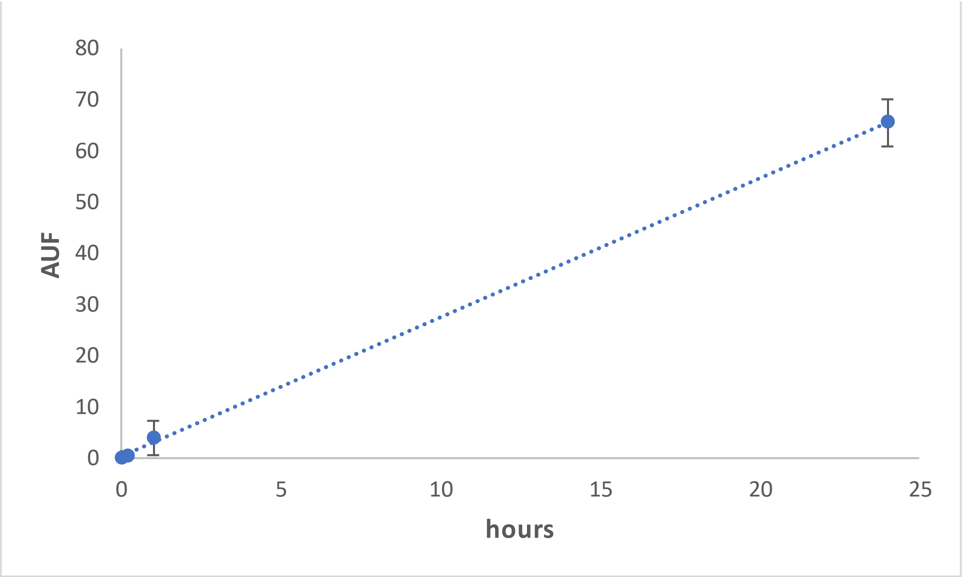

Additional file 2. Oligomerization of Aβ42 peptide after dilution to PBS buffer at 4°C for 24 hours, measured by arbitrary units of fluorescence (AUF) after staining with Thioflavin-T.
