# Supplementary material for: Echinometra lucunter molecules reduce Aβ42-induced neurotoxicity in SH-SY5Y neuron-like cells: effects on disaggregation and oxidative stress
Source: J Venom Anim Toxins Incl Trop Dis. 2023 Dec 1;29:e20230031. doi: 10.1590/1678-9199-JVATITD-2023-0031 (PMC10694836; doi:10.1590/1678-9199-JVATITD-2023-0031)
Supplement: Additional file 3. [file 1678-9199-jvatitd-29-e20230031-s3.pdf]

J Venom Anim Toxins incl Trop Dis

https://doi.org/10.1590/1678-9199-JVATITD-2023-0031

Supplementary Material to "*Echinometra lucunter* molecules reduce Aβ42-induced neurotoxicity in SH-SY5Y neuron-like cells: effects on disaggregation and oxidative stress"

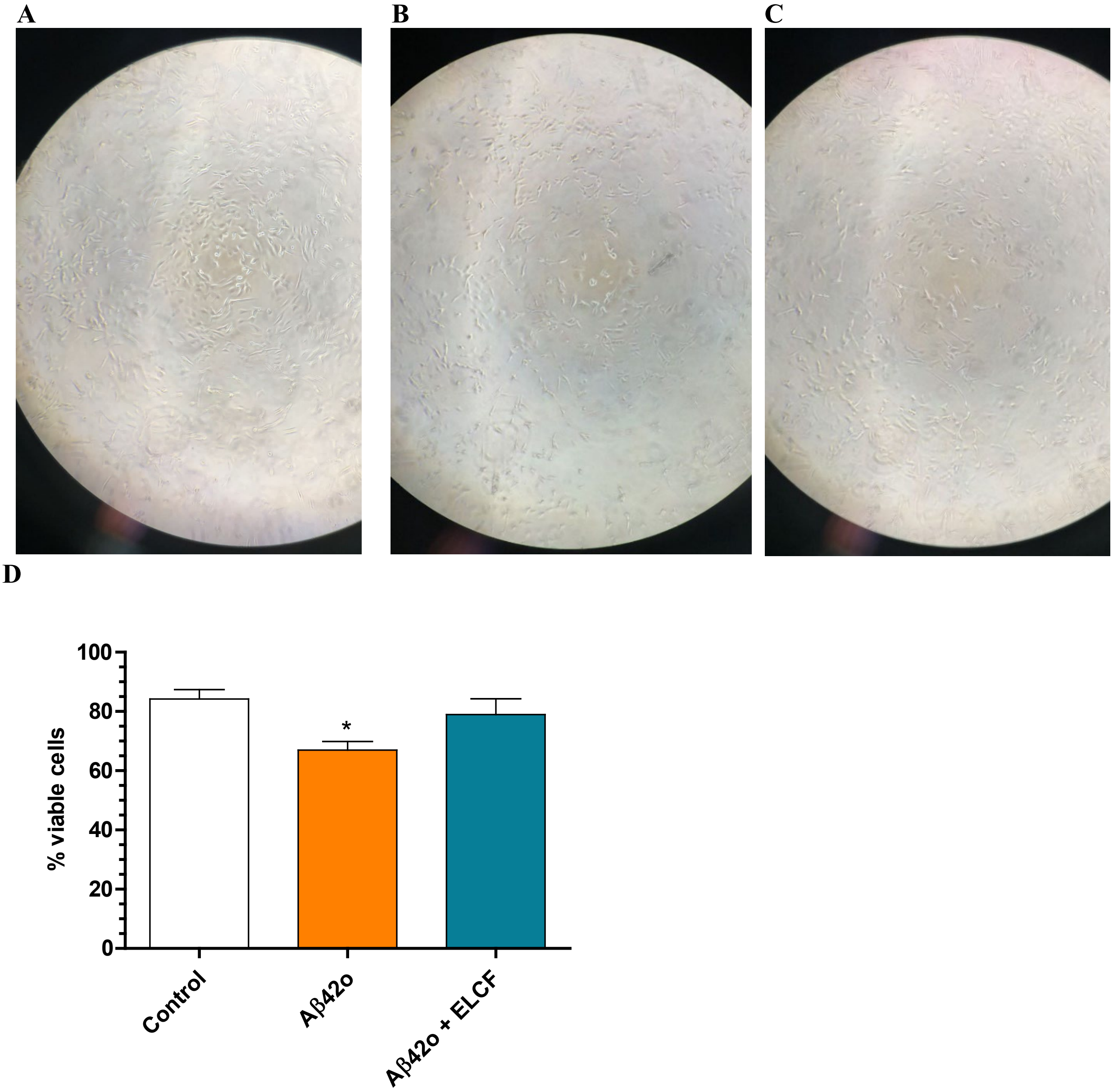

Additional file 3. Representative images of differentiated SH-SY5Y cell culture (A) without any treatment, (B) after incubation of 5 μM of Aβ42o and (C) after treatment with ELCF of cells exposed to 48h Aβ42o. The image suggests a reduction in the number of cells after treatment with both Aβ42o and ELCF, the number of cells was reduced compared to control. However, in B the morphology of cells is altered, in agreement with the MTT assay. In D, trypan blue staining of cells for counting and determination of viable cells.
