# Supplementary material for: Echinometra lucunter molecules reduce Aβ42-induced neurotoxicity in SH-SY5Y neuron-like cells: effects on disaggregation and oxidative stress
Source: J Venom Anim Toxins Incl Trop Dis. 2023 Dec 1;29:e20230031. doi: 10.1590/1678-9199-JVATITD-2023-0031 (PMC10694836; doi:10.1590/1678-9199-JVATITD-2023-0031)
Supplement: Additional file 4. [file 1678-9199-jvatitd-29-e20230031-s4.pdf]

J Venom Anim Toxins incl Trop Dis

https://doi.org/10.1590/1678-9199-JVATITD-2023-0031

Supplementary Material to "*Echinometra lucunter* molecules reduce Aβ42-induced neurotoxicity in SH-SY5Y neuron-like cells: effects on disaggregation and oxidative stress"

A

Control

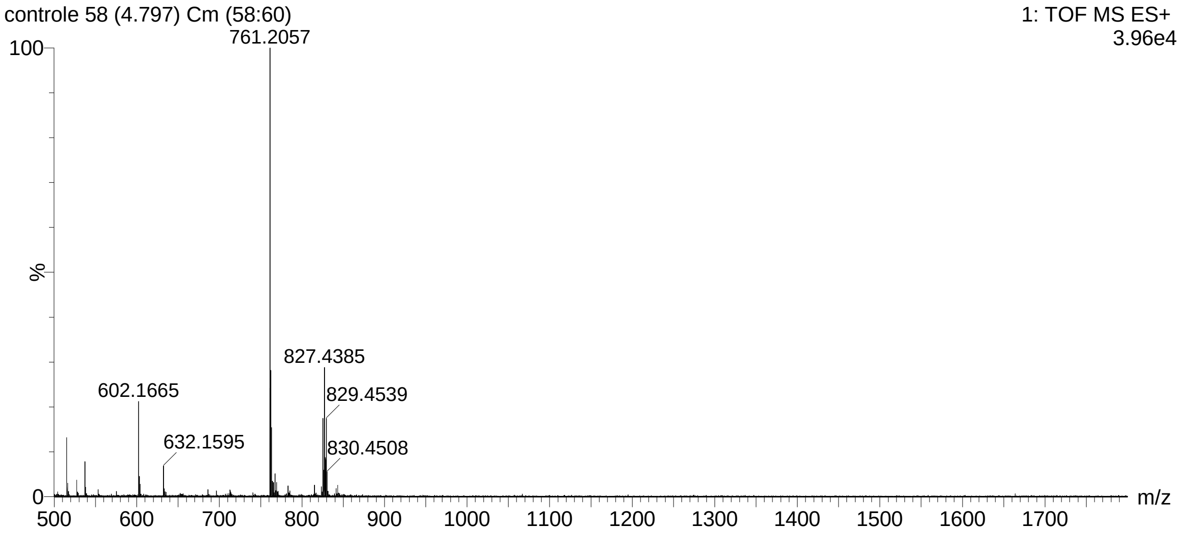

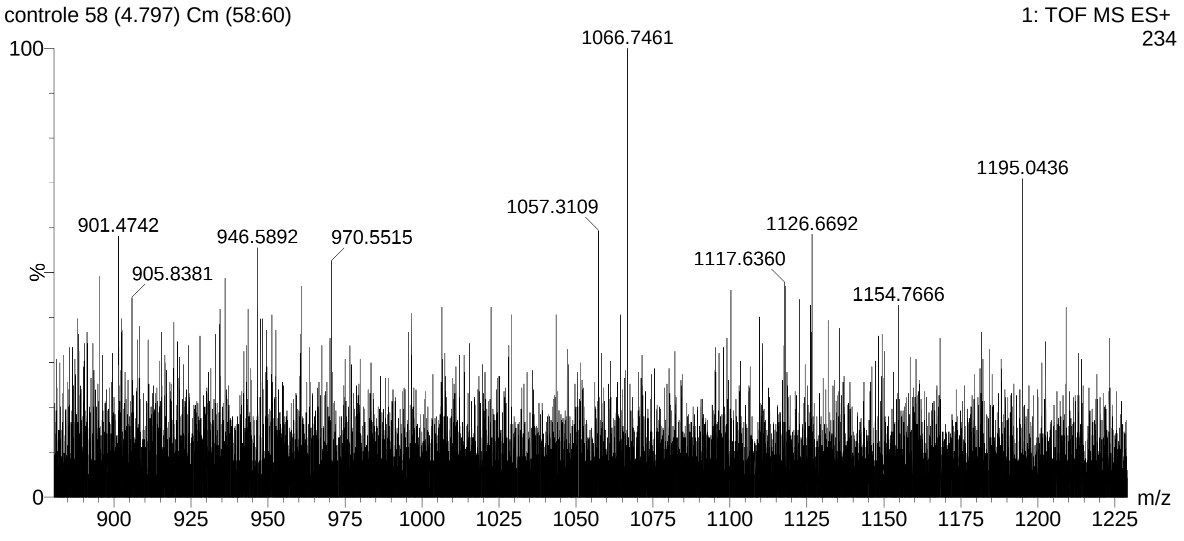

B

Aβ42

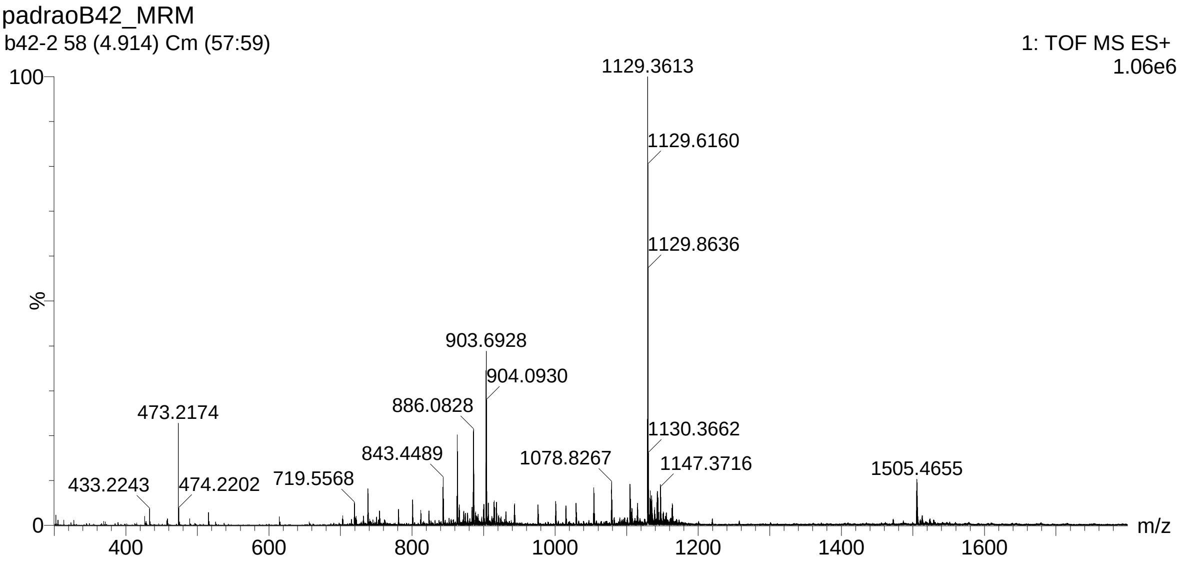

C

ELCF – prevention approach

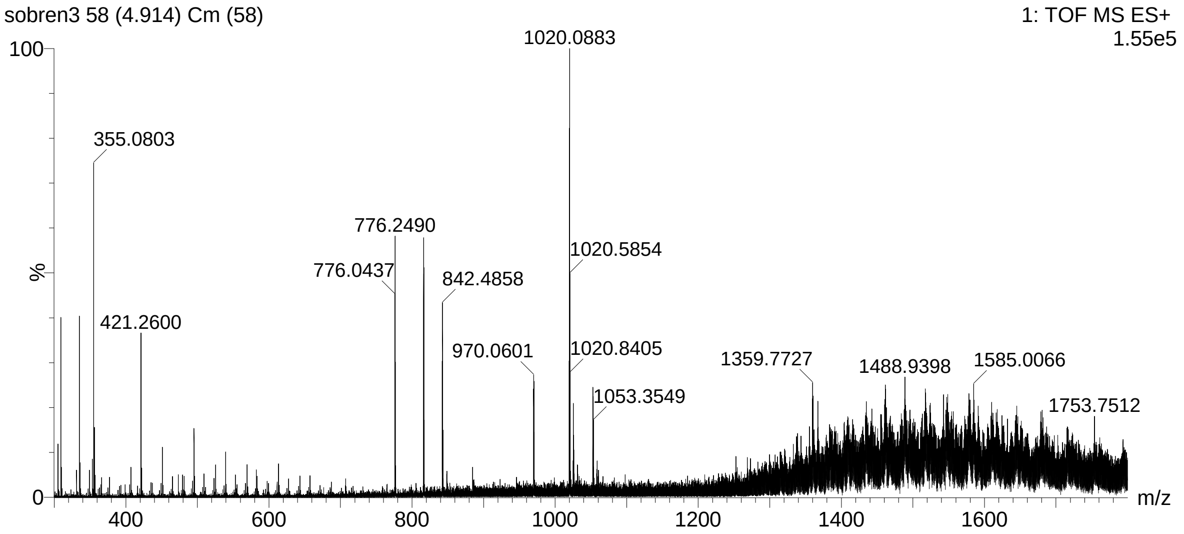

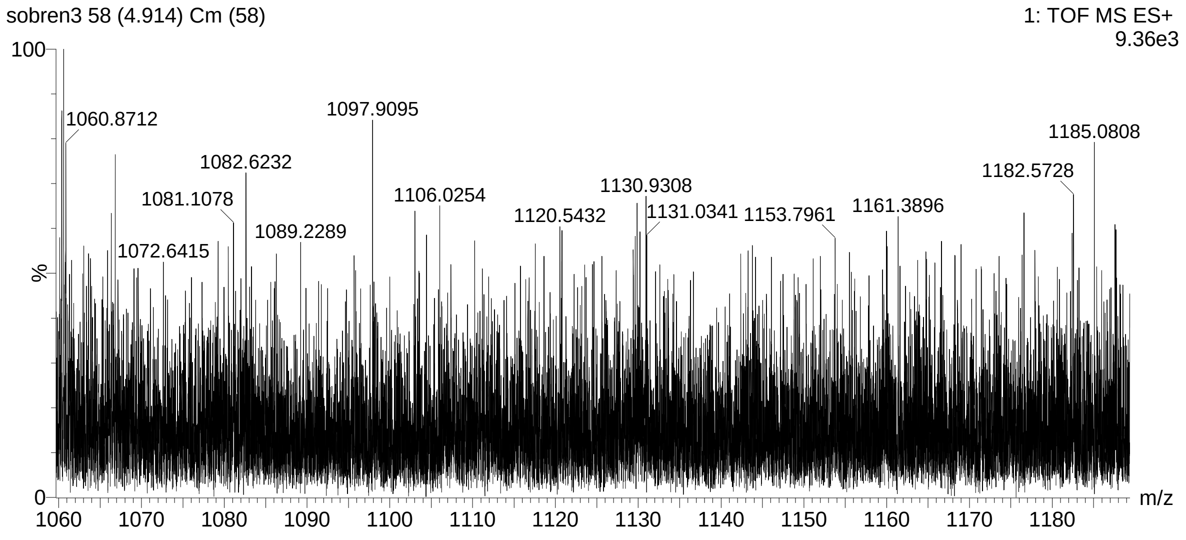

D

ELCF – treatment approach

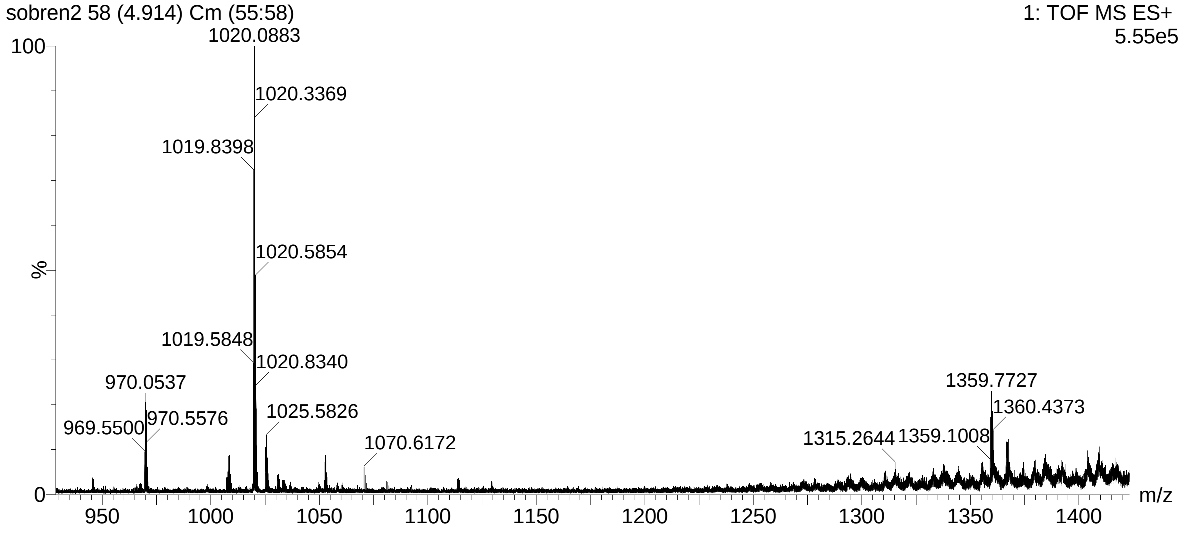

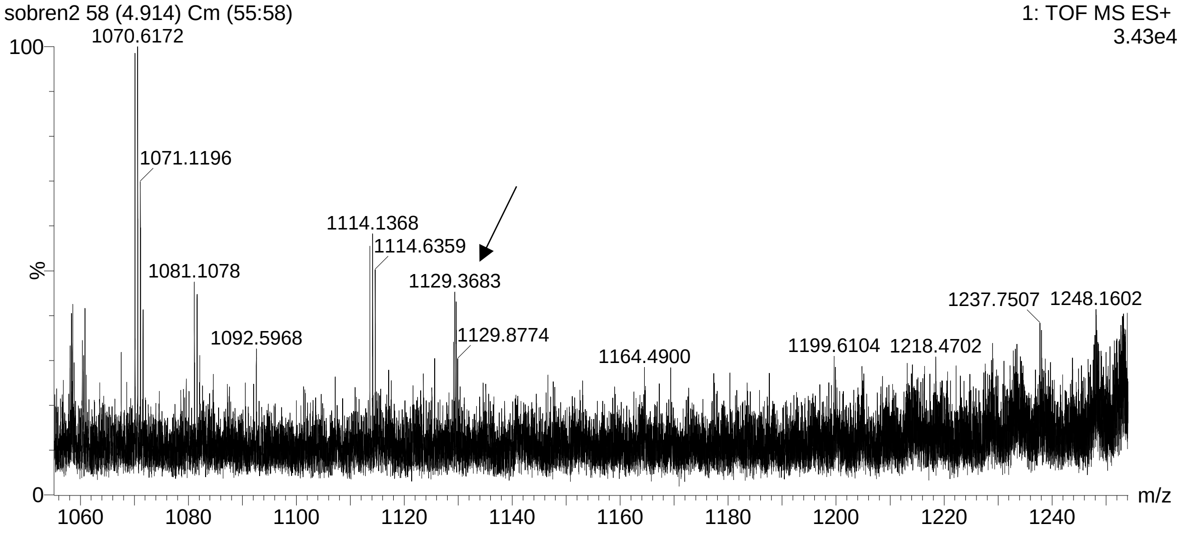

Additional file 4. Mass spectra of culture media from SH-SY5Y cell culture. (A) Control, without treatment; (B) cells treated with 5 µM Aβ42o, where is possible to see ions 1129 and 1505 m/z, correspondent to the amyloid peptide; (C) cells treated with ELCF and then 5 µM Aβ42o (prevention approach), where no Aβ42o ions were detected; (D) cells treated with 5 µM Aβ42o and then ELCF (treatment approach), where is possible to see the ion 1129 m/z (arrow in the zoomed image).
